# Studying local Berry curvature in 2H-WSe_2_ by circular dichroism photoemission utilizing crystal mirror plane

**DOI:** 10.1038/s41598-020-79672-6

**Published:** 2021-01-18

**Authors:** Soohyun Cho, Jin-Hong Park, Soonsang Huh, Jisook Hong, Wonshik Kyung, Byeong-Gyu Park, J. D. Denlinger, Ji Hoon Shim, Changyoung Kim, Seung Ryong Park

**Affiliations:** 1 State Key Laboratory of Functional Materials for Informatics, Shanghai Institute of Microsystem and Information Technology (SIMIT), Chinese Academy of Sciences, Shanghai, 200050 People’s Republic of China; 2 Center for Correlated Electron Systems, Institute for Basic Science (IBS), Seoul, 08826 Republic of Korea; 3 CAS Center for Excellence in Superconducting Electronics (CENSE), Shanghai, 200050 People’s Republic of China; 4 Department of Physics, Sungkyunkwan University, Suwon, 16419 Republic of Korea; 5 Department of Physics and Astronomy, Seoul National University (SNU), Seoul, 08826 Republic of Korea; 6 The Molecular Foundry, Lawrence Berkeley National Laboratory, Berkeley, CA 94720 USA; 7 Pohang Accelerator Laboratory, Pohang University of Science and Technology, Pohang, 37673 Republic of Korea; 8 Advanced Light Source, Lawrence Berkeley National Laboratory, Berkeley, CA 94720 USA; 9 Department of Chemistry, Pohang University of Science and Technology, Pohang, 37673 Republic of Korea; 10 Department of Physics and Division of Advanced Nuclear Engineering, Pohang University of Science and Technology, Pohang, 37673 Republic of Korea; 11 Department of Physics, Incheon National University, Incheon, 22012 Republic of Korea

**Keywords:** Two-dimensional materials, Electronic structure, Electronic properties and materials

## Abstract

It was recently reported that circular dichroism in angle-resolved photoemission spectroscopy (CD-ARPES) can be used to observe the Berry curvature in 2H-WSe_2_ (Cho et al. in Phys Rev Lett 121:186401, 2018). In that study, the mirror plane of the experiment was intentionally set to be perpendicular to the crystal mirror plane, such that the Berry curvature becomes a symmetric function about the experimental mirror plane. In the present study, we performed CD-ARPES on 2H-WSe_2_ with the crystal mirror plane taken as the experimental mirror plane. Within such an experimental constraint, two experimental geometries are possible for CD-ARPES. The Berry curvature distributions for the two geometries are expected to be antisymmetric about the experimental mirror plane and exactly opposite to each other. Our experimental CD intensities taken with the two geometries were found to be almost opposite near the corners of the 2D projected hexagonal Brillouin zone (BZ) and were almost identical near the center of the BZ. This observation is well explained by taking the Berry curvature or the atomic orbital angular momentum (OAM) into account. The Berry curvature (or OAM) contribution to the CD intensities can be successfully extracted through a comparison of the CD-ARPES data for the two experimental geometries. Thus, the CD-ARPES experimental procedure described provides a method for mapping Berry curvature in the momentum space of topological materials, such as Weyl semimetals.

## Introduction

Angle-resolved photoemission spectroscopy (ARPES) is used to directly measure the band structure of solids and is an essential experimental tool for solid state physics research^1–3^. In addition to the band structure, ARPES provides information on other aspects of the electronic structure. For example, ARPES with a spin detector can be used to obtain spin information of the initial states^4–7^. Polarization dependent experiments can provide symmetry information on the initial states; initial states from, for example, $p_x$ and $p_y$ orbitals can show dramatically different ARPES intensities depending on the polarization of the incident light^1,8^.

In recent years, there has been much interest in using circular dichroism (CD) in ARPES as a way to measure some aspects of initial states, such as the orbital angular momentum (OAM)^9,10^ or the Berry curvature^11^. It is well understood that OAM plays an important role in spin-split phenomena in systems without inversion symmetry^12–16^, such as surfaces of solids and monolayer (ML) transition metal dichalcogenides^17–19^. CD-ARPES has been utilized to obtain the crucial information on the electronic structures of such systems^12,16,20,21^. While the final state of the photoemission process certainly has an effect on the CD-ARPES intensities^22–24^, experimental results show that CD-ARPES is a rough measure of the OAM of the initial state^22–24^ if the photon energy is not too low^25^.

Exploiting this feature in CD-ARPES measurements, information on the OAM and hidden Berry curvature of 2H-WSe_2_ was recently obtained using CD-ARPES^26^. An important aspect of this research was that the Berry curvature (or OAM) contribution to the CD-ARPES intensity could be isolated by decomposing the CD-ARPES intensity map into symmetric and antisymmetric components about the experimental mirror plane, which is perpendicular with respect to the crystal mirror plane of 2H-WSe_2_. The symmetric component was attributed to the OAM or Berry curvature contribution, since the electronic structure should be symmetric about the chosen experimental mirror plane set along *K*–$\Gamma$–$K'$ in momentum space^26^.

## Results

**Figure 1:**
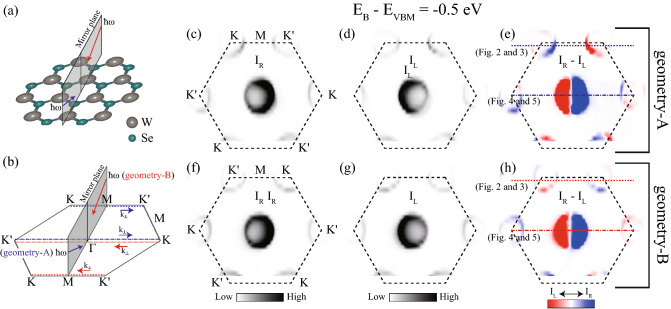
(**a**) Crystal structure of 2H-WSe_2_ showing the top atomic layer of the bulk crystal. W and Se atoms are shown as gray and green balls, respectively. The experimental mirror plane is the same as the crystal mirror plane in this experimental geometry. (**b**) Experimental geometry with a hexagonal Brillouin zone (BZ). The mirror plane indicates the experimental mirror plane made by the normal direction of the sample surface and the incident light direction. Blue (*geometry-A*) and red (*geometry-B*) arrows on the mirror plane indicate the direction of the incident light. $k_{\perp }$ indicates the momenta of the angle-resolved photoelectron spectroscopy (ARPES) cut, the main focus of this work. ARPES cut data along the directions shown as blue (red) lines are taken with the incident light indicated by blue (red) arrows. The same mirror plane was set for both cases. ARPES intensity maps at a constant binding energy are shown in (**c**) with right circularly polarized (RCP) light coming from the direction indicated as a blue arrow, in (**d**) with left circularly polarized (LCP) light coming from the direction indicated as a blue arrow, in (**f**) with RCP coming from the direction indicated as a red arrow, and in (**g**) with LCP coming from the direction indicated as red arrow. (**e**) Circular dichroism (CD) obtained from the difference between (**c**) and (**d**). (**h**) CD obtained by the difference between (**f**) and (**g**).

Experimental geometry, including single crystal orientation, is especially important in this experiment. The crystal structure of the top atomic layer or ML of 2H-WSe_2_ is a hexagonal lattice, as shown in Fig. 1a; there is a unique mirror plane in the crystal structure, as indicated in the figure. The experimental mirror plane is defined by the plane defined by the normal of the sample surface and the direction of incident light. The experimental mirror plane was set to be the same as the crystal mirror plane. Two experimental geometries are possible, according to the direction of incident light, as indicated by blue and red arrows in Fig. 1a. The experimental geometries using incident light described by blue and red arrows are regarded as *geometry-A* and *geometry-B* for convention, respectively. Notably, the signals from the top layer of bulk 2H-WSe_2_ dominate the CD-ARPES data due to the surface sensitivity of ARPES^5,26–29^; the corresponding momentum space view is shown in Fig. 1b. The mirror plane is oriented along the *M*–$\Gamma$–*M* direction, and the direction of incident light is indicated by blue and red arrows on the mirror plane in Fig. 1b. This experimental geometry differs from that used in previous work^26^, in which the experimental mirror plane was rotated by 30° with respect to the crystal mirror plane, such that the experimental mirror plane is oriented along the *K*–$\Gamma$–$K'$ direction.

We expanded on our previous CD-ARPES work on 2H-WSe_2_ by focusing on a different mirror plane. Here, we report our CD-ARPES studies on 2H-WSe_2_ with the experimental mirror plane parallel to the crystal mirror plane (Fig. 1a) or along the *M*–$\Gamma$–*M* direction in momentum space (Fig. 1b). Within the experimental constraint, there are two possible experimental geometries based on the incident beam directions, as shown by the blue and red arrows in Fig. 1a,b. The CD-ARPES values for the two geometries are nearly opposite to each other near the Brillouin zone (BZ) corner, whereas they are almost identical near the $\Gamma$ point. These observations are well explained by accounting for the Berry curvature (or OAM) contribution to CD-ARPES. Our results thus indicate that the deviation from the median value between the two experimental geometries can be interpreted as the Berry curvature or OAM.

Figure 1c,d present the constant energy ARPES maps taken by RCP and by LCP incident light in *geometry-A*, respectively. The binding energy ($E_B$) of all maps shown in Fig. 1 is 0.5 eV lower than the valence band maximum energy ($E_{VBM}$). CD signals, in which the intensity corresponds to the difference in the intensity taken by RCP ($I_R$) and that taken by LCP ($I_L$), are mapped in the momentum space (Fig. 1e). The antisymmetric function of the CD map for the experimental mirror plane is expected for this experimental geometry, given that the Berry curvature (or OAM) is also antisymmetric with regard to the experimental geometry. Figure 1f–h present the ARPES maps taken with RCP and LCP incident light in *geometry-B* and the corresponding CD map, respectively; the upper left corner corresponds to the $K'$ point in Fig. 1f–h and the *K* point in Fig. 1c–e. Remarkably, the CD signals at each corner of the BZ in Fig. 1h are almost opposite to those in Fig. 1e, whereas the CD signals near the center of the BZ are nearly the same. This can be explained by taking the Berry curvatures (or OAM) into account, given that the Berry curvatures (or OAM) are opposite at the *K* point and $K'$ point, whereas the Berry curvatures (and OAM) are nearly zero around the $\Gamma$ point. A detailed analysis of CD data was performed for ARPES cut data along the *K*–*M*–$K'$ and $K'$–$\Gamma$–*K* directions in *geometry-A* (blue lines in Fig. 1e) and along the $K'$–*M*–*K* and *K*–$\Gamma$–$K'$ directions in *geometry-B* (red lines in Fig. 1h).

**Figure 2:**
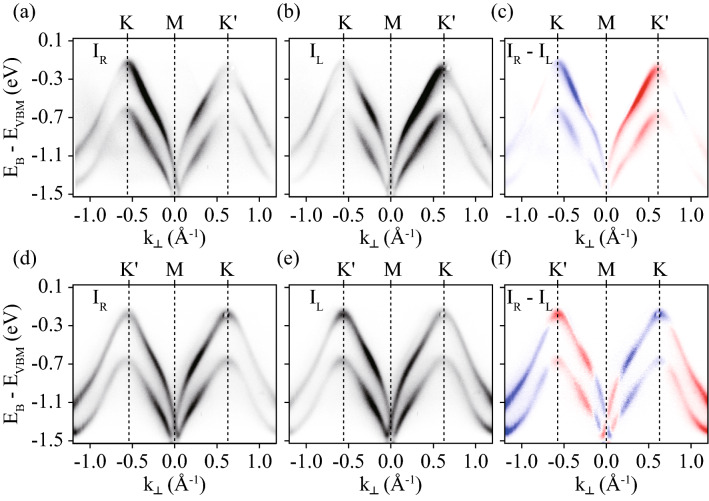
ARPES spectra taken by RCP (**a**) and LCP (**b**) light in *geometry-A* along *K*–*M*–$K'$ indicated by a dotted line in Fig. 1e. (**c**) CD-ARPES spectra obtained by the difference between (**a**) and (**b**). ARPES spectra taken by RCP (**d**) and LCP (**e**) light in *geometry-B* along $K'$–*M*–*K*, as indicated by the dotted line in Fig. 1h. (**f**) CD-ARPES spectra obtained by the difference between (**d**) and (**e**).

Figure 2a,b present ARPES spectra taken by RCP and LCP light, respectively, in *geometry-A* along *K*–*M*–$K'$, as indicated by the dotted line in Fig. 1e. Figure 2d,e present ARPES spectra taken by RCP and LCP light, respectively, in *geometry-B* along the $K'$–*M*–*K* direction, as indicated by the dotted line in Fig. 1h. Two parallel dispersive bands are evident in the spectra, of which the maxima are located at *K* and $K'$. The energy difference between the upper and lower bands originates from atomic spin–orbit coupling of the W atom^17–19^. The spin directions of the two bands are opposite, but the Berry curvature and OAM are the same, as expected from the massive Dirac–Fermion model^17–19^. ARPES intensity clearly depends on the polarization of the incident light. Figure 2c,f present CD-ARPES intensity distributions for *geometry-A* along *K*–*M*–$K'$ and for *geometry-B* along $K'$–*M*–*K*, respectively. The CD intensities of the two bands are similar at each momentum point, but the intensities are almost opposite between the CD for *geometry-A* and that for *geometry-B*; this is consistent with the constant energy maps shown in Fig. 1e,h.

**Figure 3:**
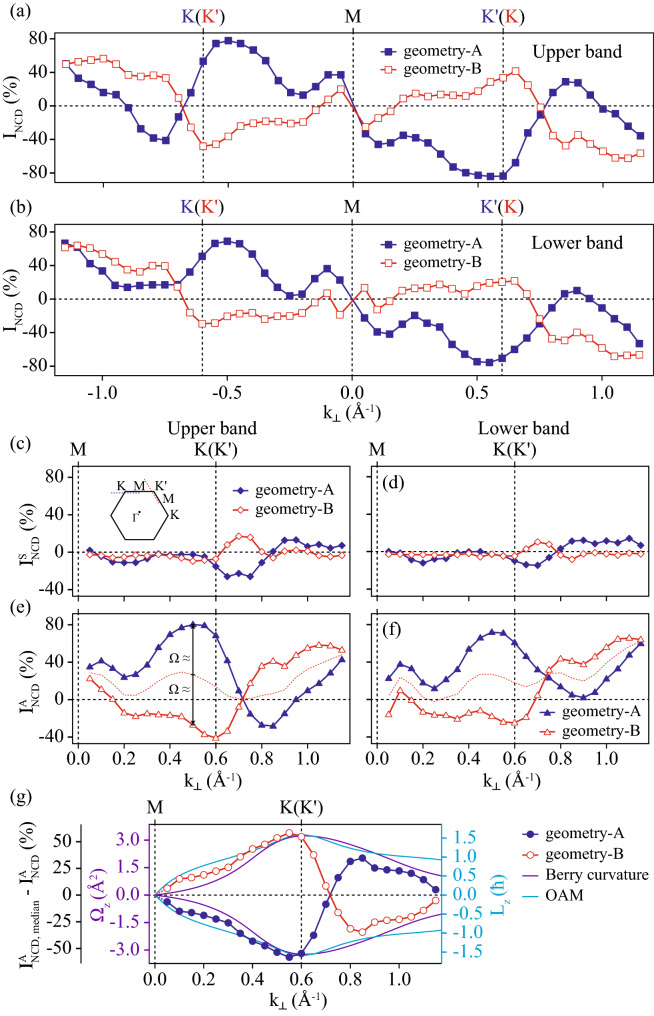
(**a**) Normalized CD intensities ($I_{NCD}$) from the upper band as a function of momenta along *K*–*M*–$K'$ (blue filled squares) and along $K'$–*M*–*K* (red empty squares). (**b**) The same as (**a**) but from the lower band. (**c**,**e**) Symmetric ($I^S_{NCD}$) and anti-symmetric components ($I^A_{NCD}$) of $I_{NCD}$ from the upper band, respectively. (**d**,**f**) Symmetric and anti-symmetric components of $I_{NCD}$ from the lower band, respectively. The dotted lines in (**e**) and (**f**) indicate the median values of $I^A_{NCD}$ between the two direction cuts. (**g**) Difference between $I^A_{NCD}$ from the upper band along each direction and the median values with theoretically calculated Berry curvature and OAM.

Normalized CD intensities ($I_{NCD}$) as a function of momentum are shown in Fig. 3a for the upper band and in Fig. 3b for the lower band. $I_{NCD}$ is obtained by ($I_R-I_L$)/($I_R+I_L$), where $I_R$ and $I_L$ correspond to the ARPES intensity taken with RCP and LCP, respectively. $I_{NCD}$ for the upper band along *K*–*M*–$K'$ in *geometry-A*, as indicated by the filled squares in Fig. 3a, has a positive value toward the *K* point from the *M* point. $I_{NCD}$ exhibits a slight sign change beyond *K* and $K'$ points, although it is difficulty to catch the fact in Fig. 2c due to very weak ARPES intensities. $I_{NCD}$ for the upper band along $K'$–*M*–*K* (*geometry-B*), indicated by the empty squares in Fig. 3a, exhibits a negative value toward the $K'$ point from the *M* point and a positive value toward the *K* point from the *M* point, except very close to the *M* point, as we can also notice in Fig. 2f; sign changes beyond $K'$ and *K* were also evident in the data. The $I_{NCD}$s in *geometry-A* and *-B* are roughly opposite, but not exactly. The $I_{NCD}$ for the lower band in *geometry-A* and *geometry-B* are also similar to those of the upper band, but they are slightly weaker.

$I_{NCD}$ consists of symmetric ($I^S_{NCD}$) and antisymmetric functions ($I^A_{NCD}$) about the experimental mirror plane (*M* point). Figure 3c,d present the $I^S_{NCD}$s for the upper and lower bands from two geometries, respectively. Figure 3e,f present the $I^A_{NCD}$s for the upper and lower bands from two geometries, respectively. As shown in the figures, the $I^S_{NCD}$s were close to zero, and $I^A_{NCD}$s were dominant components, regardless of the geometry or band. An asymmetric CD-ARPES distribution about the experimental mirror plane is a usual feature from solids^23,30,31^, as the inversion symmetry along the surface normal direction is lifted on the surface of solids, which is similar to an oriented CO molecule system^32,33^. The CD-ARPES contribution caused by the inversion symmetry breaking in the material surface can be called surface effects. However, it is surprising that the CD was nearly opposite between *geometry-A* and *-B*. Based on this finding, we believe that a substantial portion of $I^A_{NCD}$ originates from the Berry curvature (or OAM), given that the CD signs follow the Berry curvature (or OAM) direction, as shown in Figs. 1e,h and 2c,f.

**Figure 4:**
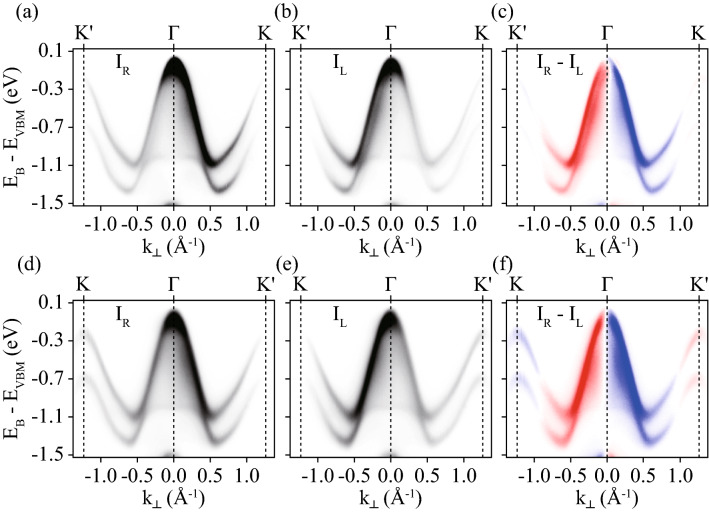
ARPES intensities taken by RCP (**a**) and LCP (**b**) light in energy and momentum space along $K'$–$\Gamma$–*K* indicated by the blue dotted line in Fig. 1e. (**c**) CD obtained by the difference between (**a**) and (**b**). ARPES intensities taken by RCP (**d**) and LCP (**e**) light in energy and momenta along *K*–$\Gamma$–$K'$, as indicated by the red dotted line in Fig. 1h. (**f**) CD obtained by the difference between (**d**) and (**e**).

**Figure 5:**
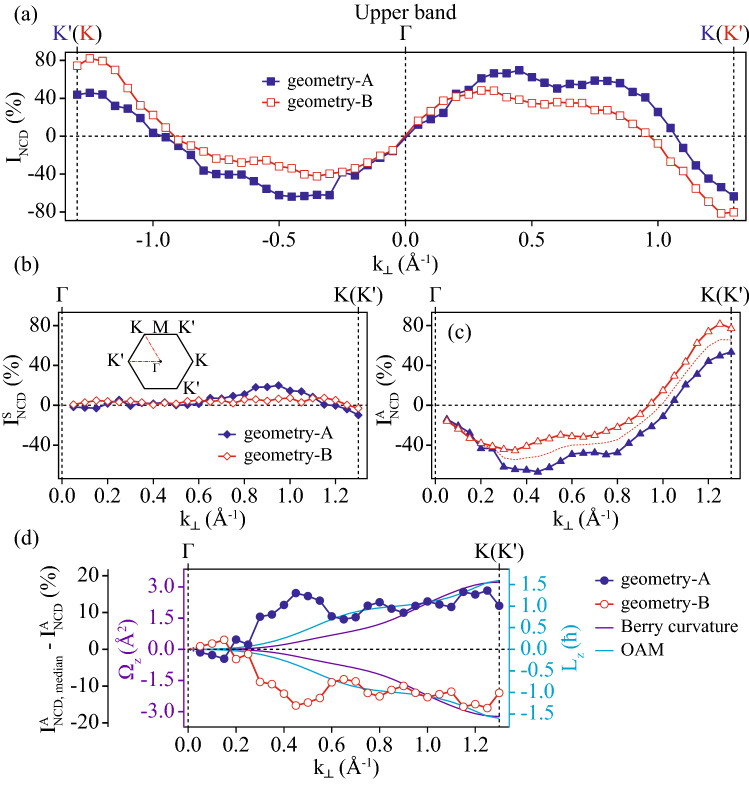
(**a**) $I_{NCD}$ along $K'$–$\Gamma$–*K* (blue filled squares) and along $K'$–$\Gamma$–*K* (red empty squares). (**b**,**c**) $I^S_{NCD}$ and $I^A_{NCD}$ of $I_{NCD}$, respectively. The dotted lines indicate the median values of $I^A_{NCD}$. (**d**) Difference between $I^A_{NCD}$ along each direction and the median values, along with the theoretically calculated Berry curvature and OAM.

It is important to isolate the Berry curvature contribution to $I^A_{NCD}$ from other contributions. The Berry curvature (or OAM) contribution to CD-ARPES should be exactly opposite between the normalized CD-intensities along *K*–*M*–$K'$ in *geometry-A* and along $K'$–*M*–*K* in *geometry-B*, because the Berry curvatures (or OAM) themselves are exactly opposite for *K* and $K'$ points. We assume that other contributions, mainly the surface effects, are the same, regardless of the geometry. Then, the median values (red dotted lines in Fig. 3e,f) of $I^A_{NCD}$s from *geometry-A* and *-B* can be considered from the other contributions to $I^A_{NCD}$s. Additionally, this assumption is experimentally justified by CD-ARPES data near the $\Gamma$ point, as shown in Figs. 4 and 5. The difference in $I^A_{NCD}$ with respect to the median value is exactly opposite between the *K*–*M*–$K'$ cut in *geometry-A* and the $K'$–*M*–*K* cut in *geometry-B*; this difference can be interpreted as the Berry curvature (or OAM) contribution to $I^A_{NCD}$. Figure 3g presents the differences, along with the theoretical values of the Berry curvature and OAM. The differences are similar to the Berry curvature and OAM, except for the crossing at zero and the changing signs near 0.7 Å$^{-1}$.

The sign change of the difference of $I^A_{NCD}$ from the median value is mainly due to the change in the final state character as the momentum of the photoelectron varies. We know that the wave function characters of the initial states near the *K*($K'$) point change gradually and depend on the distance from the *K*($K'$) point in the massive Dirac–Fermion model^17–19^. The sign of CD-ARPES data can be reversed for the same initial states by only changing the final states, as indicated in the photon energy dependence of CD-ARPES^22,23^.

Figure 4 presents the ARPES cuts and CD-ARPES data along the $K'$–$\Gamma$–*K* in *geometry-A*, and along *K*–$\Gamma$–$K'$ in *geometry-B*, as indicated in Fig. 1. These cuts are special, in terms of the Berry curvature and OAM of the electronic states near the $\Gamma$ point being almost negligible, compared with those of states near the *K*($K'$) point. Therefore, the Berry curvature contribution to CD-ARPES data is expected to be almost zero near the $\Gamma$ point. The CD-ARPES signals in both geometries are quite strong near the $\Gamma$ point and exhibit a clear node at $\Gamma$, indicating no symmetric component of the CD intensity. The CD-ARPES intensities near the *K*($K'$) point from both geometries are much weaker than those near the $\Gamma$ point, and the CD-ARPES intensities near the *K*($K'$) point from *geometry-A* are even weaker than those from *geometry-B*.

Figure 5a–c present $I_{NCD}$s, $I^S_{NCD}$s, and $I^A_{NCD}$s, respectively. The symmetric components are negligible; the asymmetric components make up the majority of the $I_{NCD}$s (Fig. 5b,c). Remarkably, $I_{NCD}$s along $K'$–$\Gamma$–*K* in *geometry-A* and along *K*–$\Gamma$–$K'$ in *geometry-B* are the same near the $\Gamma$ point (Fig. 5a), and $I^A_{NCD}$s are, in turn, the same near the $\Gamma$ point (Fig. 5c). Figure 5d presents the deviations of $I^A_{NCD}$s from the median value, along with the theoretical values of the Berry curvature and the OAM. The deviation is almost zero near $\Gamma$ point and begin to have large value at the momentum at which the Berry curvature and the OAM are also about to increase from almost zero value. This provides experimental evidence that the deviation from the median value of $I_{NCD}$s in *geometry-A* and *-B* can be interpreted as the Berry curvature (or OAM) contribution. Although the Berry curvature and the OAM continually increase as they approach the *K*($K'$) point, the deviation from the median value from CD-ARPES data seems to be almost constant away from the $\Gamma$ point.

## Discussions

Let us briefly touch upon the possible incident photon energy dependence in CD-ARPES or the final state effect. This is because one can wonder if the CD-ARPES pattern we obtained is seen only with the particular photon energy and a different photon energy may give us a different result. In such case, changing the photon energy will also change the CD-ARPES pattern and the CD-ARPES may not be related to OAM or the local Berry curvature. We would like to point out that incident photon energy dependent CD-ARPES has been performed on the same material^26^. The results showed that CD-ARPES features related to the local Berry curvature are the same regardless of the photon energy. Even though it was for a different plane of incidence compared to the current one, the photon energy independence of the pattern provides a good reason to believe that the CD-ARPES pattern is proportional to OAM or the local Berry curvature. In some the other systems such as Bi$_2$Te$_3$^24,34^, PtCoO$_2$^16^and Au(111)^25^, CD-ARPES results show a sign change. Yet, those results still show that node lines in CD-ARPES map remain the same except a special resonant channel is involved^25^. In addition, characteristic patterns of CD-ARPES map cannot be explained without consideration of OAM^25^. Therefore, we argue that the interpretation of the CD-ARPES intensity in this work should be robust although the data was taken only with a single photon energy.

CD-ARPES data on 2H-WSe$_2$ were taken with the crystal mirror plane set as the experimental mirror plane. Within the experimental constraint, there are two possible experimental geometries. We found that CD-ARPES data for the two geometries (*geometry-A* and *-B*) are almost opposite to each other near the BZ corners, and nearly the same near the $\Gamma$ point. The experimental observations are well explained by accounting for the Berry curvature (or OAM) contribution to CD-ARPES. The Berry curvature (or OAM) contribution to the $I_{NCD}$s can be quantitatively extracted through an analysis that compares $I_{NCD}$s for the two geometries. Our results provide experimental evidence that the deviation from the median value between the two experimental geometries can be interpreted as the Berry curvature or the OAM. Our work may be applicable to observations of the Berry curvature or the OAM in topological materials, such as Weyl semimetals^35–37^and Berry curvature dipole materials^38–44^.

## Methods

ARPES measurements were performed at the beam line 4.0.3 of the Advanced Light Source at the Lawrence Berkeley National Laboratory, equipped with a VG Scienta R8000 electron analyzer. The energy resolution was better than 20 meV, with a momentum resolution of 0.004 Å$^{-1}$. The degree of left- and right-circularly polarized (LCP and RCP, respectively) 94 eV light was better than 80%. Single-crystal bulk 2H-WSe$_2$ was purchased from HQ Graphene (Groningen, Netherlands); the crystal was cleaved in situ at 100 K under high vacuum conditions ($<1\times 10^{-10}$ Torr).

The normalized CD-intensity ($I_{NCD}$) is defined as the difference between the peak height of the energy distribution curve taken with RCP and LCP, divided by their sum. This normalization can be expressed as $I_{NCD}$=($I_R-I_L$)/($I_R+I_L$), as in previous papers^9,10,16,20,22,23,25,26^. From this, the symmetric and antisymmetric components can be calculated as a function of the momentum *k* as $I^S_{NCD}(k) = [I_{NCD}(-k) + I_{NCD}(k)]/2$ and $I^A_{NCD}(k) = [I_{NCD}(-k) - I_{NCD}(k)]/2$, respectively.

The momentum-dependent OAM $L_z$ of ML 2H-WSe$_2$ can be determined from density functional theory calculations. For the calculations, we adopted the structural parameters of 2H-WSe$_2$ from an experiment^45^ to construct the ML 2H-WSe$_2$ structure and removed a WSe$_2$ layer in the unit cell. We allowed for more than 20 Å spacing (vacuum) between neighboring ML 2H-WSe$_2$ layers to make the interaction between layers negligible. Calculations were performed using OpenMX package^47–48^, which is based on pseudo-atomic localized basis functions. The pseudo-atomic orbital basis was chosen to be *s*2*p*2*d*1 for both W and Se atoms. The generalized gradient approximation Perdew–Burke–Ernzerhof functional^49^ was applied. We relaxed the electronic structure with the convergence criteria of 10$^{-7}$ Hartree, using an energy cutoff of 120 Ry and a $10\times 10\times 1$ mesh for *k*-point sampling. Spin–orbit coupling was considered. Using the linear combination of atomic orbitals (LCAO) coefficients, we calculated the momentum-dependent $L_z$ values along a certain direction in the BZ.

The tight binding model was applied to obtain the Berry curvature $\Omega _z$ for the ML 2H-WSe$_2$. The parameters were fitted until the dispersion was consistent with ARPES measurements^50,51^ and previous results^18,19^. For the Berry curvature calculation, we considered a tight-binding Hamiltonian based on *d*-orbital hybridization of the W atom and the *p*-orbitals of the Se atom. The Berry curvature was then calculated using the Thouless–Kohmoto–Nightingale–den Nijs formula. We refer to a previous study^52^ in which the Berry curvature was calculated using a *k*-mesh of $80 \times 80$.
